# Religiosity, Financial Risk Taking, and Reward Processing: An Experimental Study

**DOI:** 10.1007/s10899-024-10324-4

**Published:** 2024-06-11

**Authors:** Frederique J. Vanheusden, Sundara Kashyap Vadapalli, Mamunur Rashid, Mark D. Griffiths, Amee Kim

**Affiliations:** 1 Department of Engineering, School of Science and Technology, Nottingham Trent University, New Hall Block, Room 177, Clifton Campus, Clifton Lane, Nottingham, NG11 8NS UK; 2 Christ Church Business School, Canterbury Christ Church University, Canterbury, UK; 3 International Gaming Research Unit, Psychology Department, Nottingham Trent University, Nottingham, UK

**Keywords:** Financial risk-taking, Religion, Electro-encephalography, Iowa Gambling Task, Balloon Analog Risk Task, Mixed-gambling loss-aversion task

## Abstract

The present study investigated the extent to which financial risk-taking (FRT) perspectives and religiosity influenced an individual’s performance on financial decision-making tasks under risk and/or uncertainty. It further investigated the potential to measure this interaction using electro-encephalogram (EEG) assessments through reward-related event-related potentials (P3 and FRN). EEG data were collected from 37 participants undergoing four decision-making tasks comprising the Balloon Analogue Risk Task (BART), Iowa Gambling Test (IGT), Mixed-Gamble Loss-Aversion Task (MGLAT), and MGLA-Success Task (MGLAST). The present study found that BART performance may be affected by an interaction of FRT perspectives and religiosity. The physiological effects of task feedback were also distinguished between religious and non-religious individuals objectively with EEG data. Overall, while religiosity and FRT may not significantly influence IGT and MGLA performance, and interact with BART in a complex way, physiological reaction towards feedback after BART performance appears to be strongly affected by religiosity and FRT perspectives.

## Introduction

Faith, spiritual beliefs, and religiosity form a significant cultural factor when it comes to individual and social behavior. Religiosity can be conceptualized at both an institutional and individual dimension (Kádár et al., 2023), with its institutional dimension involving an individual’s attitudes towards and engaging with religious organizations and participating in events, and its individual dimension involving the belief in deities or a higher power, affection to this higher power, and its effects on an individual’s behavior and circumstances (Kádár et al., 2023; Williams et al., 2022).

The psychosocial influence of religion in varying contexts has been reasonably established (Shatenstein & Ghadirian, 1998; Tan et al., 2014). However, the exact influence of religiosity on personal and collective attitudes and the consequent impact on behavior is less straightforward and more complex. Even the interconnection between religiosity and behavior regarding specific fundamental aspects of existence (Baier & Wright, 2001; Lawrence et al., 2016; Simpson & Ramberg, 1992) is largely inconsistent, resulting in non-significant outcomes on association. The extent and contexts under which individuals adhere to and deviate from religious norms are not yet fully understood.

### Religiosity and (Financial) Risk-Taking

In the world of financial matters, these inconsistencies become more pronounced. However, due to the prominent effect that money has on an individual – by providing visible and concretely definable incentives for behavior (Lea & Webley, 2006) – the connection between religiosity and financial attitudes and its consequent real-world outcomes, has workable implications. While there is no concrete theoretical framework to support any direct relationship between religiosity and financial attitudes/behavior, many studies have investigated possible relationships between religiosity and general risk-taking or problematic gambling (Calado et al., 2023; Kádár et al., 2023; Kanabar et al., 2024). Findings from these studies may be extrapolated to identify differences between individuals according to their likelihood of engaging with higher-risk financial investment opportunities (e.g., stock market participation).

Chan et al. (2014) reported that priming individuals with reminders of divinities encouraged risk-taking behavior (including financial risks) in contrast to those who were not primed with reminders of divinities. Kupor et al. (2015) reported similar findings. These studies hypothesized that religiosity creates psychological safety nets for individuals to ‘take a chance’ and contradict studies that have suggested that religion acts as an effective deterrent against (financial) risk-taking behaviors (Beyerlein & Sallaz, 2017), but rather deters against immoral behaviors which may involve risk-taking (Kupor et al., 2015). However, Gervais et al. (2020) were unable to replicate these results, thereby suggesting that the association between religious priming and risk-taking was not substantially pronounced.

Other studies have associated religiosity with risk aversion in different contexts. Bartke and Schwarze (2008) reported an association between religiosity, religious affiliation, and risk-taking, while reporting a non-significant association between risk-taking and nationality. Jiang et al. (2015) reported that family firms with religious founders were more risk averse as opposed to their counterparts. Wijaya et al. (2022) reported that a higher level of religiosity was associated with increased risk aversion within the same religious group. Noussair et al. (2013) reported that risk aversion was driven by social aspects of religious membership rather than religious belief, while confirming the association between religiosity, risk aversion, and religious affiliation.

From a problematic gambling perspective, various studies have shown opposite effects of religiosity on problematic gambling, both from the institutional and individual dimension of religiosity. From an institutional dimension, Lam (2006) showed that individuals who participate less frequently in religious activities are more likely to engage in gambling activities. However, Toneatto (1999) indicated that adhering to religious beliefs or performing specific religious practices could increase faith in winning, and therefore increase the likelihood of gambling.

Other studies indicate that the religious aspects of attending religious services can reduce the likelihood of gambling, as these services often portray gambling and other financial risk-taking behaviors as sinful (Beyerlein & Sallaz, 2017; Casey et al., 2011; Uecker & Stokes, 2016), suggesting that the institutional aspects of religion (such as group identity and norms) may also influence attitudes towards gambling, as seen in other contexts (Williams et al., 2017). Another institutional factor may be the religious affiliation of an individual, with some religions considering gambling sinful, and other religions not bearing obvious moral sanctions against gambling (Calado et al., 2023).

However, there are significant exceptions that further complicate general inferences regarding the relationship between religious affiliation and financial risk-taking (Mutti-Packer et al., 2017). Some religions condemn gambling but will tolerate lottery-based activities where money spent by participants is returned to participants (with a possible gain), or where the profit made by the lottery is used to support the community (Caple & Roddy, 2023; Hassanat & Al Tarawneh, 2015). For participants, these initiatives can blur the lines between what is excessive financial risk-taking (gambling) and investing in communities or commodities. In extreme cases, this may lead to individuals investing in excessive commodities for the profit of pastors or other members of the religious community (Udechukwu, 2021).

Some financial risk-taking behaviors comparable to gambling are also theologically sanitized (Coleman, 2016). Moreover, some religions may not provide explicit guidance on forms of financial risk-taking (e.g., purchasing a luxury car) that could lead to severe losses for a family, leading members of the religious community to follow global trends in these decisions (Arli et al., 2020; Nwankwo et al., 2014). The relationship between religion and institutional norms within religions and their influence on the morality and behavior of individuals is ultimately not straightforward.

From an individual dimension, studies examining religiosity and problematic gambling have shown mixed findings (Kanabar et al., 2024). While spirituality has shown protective elements and positive contributions towards recovery from substance abuse (Kádár et al., 2023), other studies showed that a religious aspect in recovery therapy may reduce the likelihood of problem gamblers to engage with therapy (Schuler et al., 2016). Increased religiosity has also been shown to increase the likelihood to participate in gambling activities (Williams et al., 2022). This is often explained through the illusion of control framework (Browne et al., 2019; Calado et al., 2023), with problem gamblers believing that with the help of divine or spiritual interventions, they can control gambling outcomes (Toneatto, 1999). A further complication is the aspect of religious struggles and loss of faith, where individuals who are struggling with their religion may become more prone to engage with activities described as sinful within their religious framework, including gambling (Grant Weinandy & Grubbs, 2021).

Another aspect of the individual dimension of religiosity and wealth-seeking behavior is from the standpoint of reciprocal altruism. This refers to a behavior that enables individual organisms to indulge in behaviors that are not immediately advantageous (cost incurrence), in expectation of a potential advantage (recompense) in the foreseeable future (Trivers, 1971).

While it is difficult to arrive at a cost-benefit balance on an evolutionary basis, reciprocal altruism serves as an attempt to explain a range of behavioral conundrums such as symbiosis and social cooperation – particularly in non-human primates and humans (Kurzban et al., 2015; Trivers, 2006) – which in turn may help understand specific decisions under risk. Bulbulia (2004) hypothesized that religion mediates between social cohesion and reciprocal altruism, thereby explaining ‘costly’ religious behaviors (such as pilgrimage, donations) that are economically disadvantageous to an individual. A direct example that bridges the worlds of faith and finance is prosperity gospel, a religious concept which is based on the idea that monetary donations to a divine object will result in monetary rewards through divine sanction (Bowler, 2018).

Hobson et al. (2021) identified an interplay of factors culminating in the high-risk decision outcome among individuals who engage with prosperity gospel. These include heightened optimistic bias, high arousal positive effect, and ‘financial risk-taking’ – all cognitive processes repeatedly associated with gambling (a financial risk-taking activity) which have been reported in several studies (Cummins et al., 2009; Delfabbro, 2004). However, reciprocal altruism alone may not be a sufficient causal link for theorizing an association between financial risk-taking (FRT) and religiosity. Other associations have been explored which suggest a combination of interrelated factors.

In summary, whichever framework developed for linking religion and religiosity with (financial) risk-taking or gambling is considered, studies investigating these associations have shown inconsistent results (Kanabar et al., 2024). These inconsistent findings may be due to how constructs of religiosity rather than religiosity itself may interact with attitudes and behaviors associated with FRT. This notion is supported by studies by Williams et al. (2022) and Mónico and Alferes (2022), which suggest that approaches to religious faith can influence FRT behaviors. Therefore, it is worth considering that religious belief, as a standalone factor itself, may not be very helpful in understanding the morality of risk-based decisions. This ultimately may help explain the largely inconsistent results across different studies and challenge the rationale of grouping participants under a single defining category of subscribing to religious faith, to analyze the extent towards which religiosity and (financial) risk-taking are associated. Therefore, it may not be meaningful to suggest that all religious individuals have the same risk-taking approach or are influenced by religiosity in the same way while taking a decision under risk. Rather, it may be useful to explore how approaches to religiosity influence risk-taking, and how this relationship is further influenced by factors such as optimism bias and other environmental factors such as the institutional effects of religion – which also vary across religious populations (Saroglou & Cohen, 2013).

### Confounding Factors in Religiosity and Financial Risk-Taking

Factors such as socioeconomic drives, culture, history of FRT behaviors, and neurobiological factors could add further context to what makes individuals susceptible to risky investments or vulnerable to delayed gratification. Such factors may ultimately aid the investigation of the association between religiosity and financial risk-taking. For instance, the COVID-19 pandemic influenced gambling prevalence around the world, indicating most gamblers maintaining or decreasing their gambling frequency, but an increase in problem gambling (Brodeur et al., 2021), although most studies in the review collected self-report data. Another study showed a similar increase in gambling among individuals with gambling disorders and younger adults, but a reduction in gambling during periods of lockdown (Quinn et al., 2022). However, contrasting findings were reported in studies using actual tracking data supplied by gambling operators (Auer et al., 2023; Auer & Griffiths, 2022). These studies showed that gambling among the heaviest gamblers reduced during the initial months of the pandemic. While extant literature on the topic is not sufficiently exhaustive to establish definitive conclusions, the issue of the COVID-19 pandemic and gambling is multifaceted. This has a basis in the socioeconomic argument including the economic pressures and rising inflation amplified by the pandemic, as well as cognitive factors with some studies arguing that lockdowns increased gambling prevalence among problem gamblers through the escapism and excitement offered by online FRT activities during a time of relative social isolation (Håkansson et al., 2020).

On the other hand, sociocultural (and sometimes economic and political) factors have a visible and observable impact on religious beliefs and experiences, especially regarding the transmission and development of religious beliefs and experiences (Edgell, 2012). However, these factors do not fully explain the underlying mechanisms contributing to the neurobiological aspects of religiosity and its subsequent impact on cognition, behavior, decision-making, and mental health. The second challenge is to identify an objective predictor that explains financial risk-taking sufficiently, and then evaluate these measures between cohorts exercising different levels of religiosity. Ideally, the level of religiosity would also be evaluated using objective markers. These objective evaluations can either be achieved through observation when performing relevant tasks and/or collection of neurophysiological data.

### Physiological Measurements of (Financial) Risk-Taking and Religiosity

To date, most studies with physiological measures have collected data using either functional magnetic resonance imaging (fMRI, e.g., Miller et al. [2019]) or EEG while participants perform a risk-related task (Balconi & Angioletti, 2022; Giustiniani et al., 2019; Pornpattananangkul et al., 2019; Schmidt et al., 2018). A study by Giustiniani et al. (2019) found that asymmetry in theta-activity between the right and left hemispheres may reflect an individual’s motivation and ability to explore strategies when performing the BART. They did not find a similar correlation using the IGT.

Pornpattananangkul et al. (2019) investigated P3 response and delta-band activity after each of 120 trials of a mixed-gamble loss-aversion task (MGLAT; Tom et al. [2007]). The study found that expected utility affected P3 and delta-band activity earlier compared to utility distance, indicating that individuals will develop decision-making strategies based on motivation earlier compared to those based on conflict. However, no information about religiosity was provided for the those participating in the study, yet this could be a potential factor to determine at what time point (i.e., after how many MGLA tasks) conflict-related decision-making components start affecting EEG activity.

In a study evaluating the effect of general anxiety levels on decision-making, Schmidt et al. (2018) measured frontal midline theta (FMT) activity among 20 high and 20 low-anxious individuals performing a risk game (card selection with different odds of success). They reported that individuals with high anxiety played less riskily and had stronger FMT activity compared to low anxiety individuals. This suggested that higher anxiety is related to a higher exertion of cognitive control during risky decision-making tasks. In another study, Balconi and Angioletti (2022) showed that N2 event-related potential (ERP) amplitudes correlated strongly with Go/NoGo task performance among individuals with higher risk of internet addiction when being exposed to addiction-related background pictures.

There is growing evidence to suggest that some regions in the brain, in addition to some neural interconnections, contribute to religiosity or religious experience (Rim et al., 2019). While these regions and interconnections bear other fundamental expressions of human neural functionality, evidence suggests that religious faith is correlated with unique neural activity and in some instances, causally linked. Holbrook et al. (2016) reported that the posterior medial frontal cortex (pMFC) helps integrate information from interconnected regions of the brain and strengthen an individual’s commitment to their religious beliefs and group values when they encounter ideological conflicts or threats. Additionally, the study reported that these beliefs are vulnerable to targeted neuromodulation through transcranial magnetic stimulation. Holbrook et al. (2020) further reported reduced religious belief among participants with a downregulated pMFC when reminded of a relevant threat (such as death), but not under threat-neutral conditions. Therefore, the pMFC may mediate a threat-response to conflict stimuli, and empirical research examining the issue may be supported by evolutionary perspectives (such as reciprocal altruism and group cooperation).

A study by Tenke et al. (2017) evaluated religiosity and spirituality in a cohort over a 20-year period and reported (i) early (i.e., childhood) initiations into religiosity with higher EEG alpha readings (posterior resting alpha activity), (ii) change in religious denomination with lower EEG alpha readings, and (iii) no correlation of EEG alpha with later initiations into religiosity. Neurobiological evidence has also shown that religion may act as a potential deterrent against some manifestations of depressive disorders (Tenke et al., 2017), with posterior alpha activity also being related to better outcome in depression treatment (Bruder et al., 2008).

Religion may also be associated with differences in anatomy such as increased cortical thickness and decreased default mode network (DMN) connectivity (Rosmarin et al., 2022). However, it is important to note that the precise roles these correlates play are not fully understood. Therefore, there is insufficient evidence to link neurobiology directly with religion. Moreover, recent studies collecting EEG data in eyes-closed resting state have shown a correlation between increased theta-activity and religious coping with stress (Imperatori et al., 2020).

### Assessment of Financial Risk-Taking Preferences and Performance

Several experimental tasks have been developed to assess (financial) risk-taking preferences and behavior. Most notably, financial risk-taking behavior and the ability to develop risk-taking strategies has been evaluated using the Iowa Gambling Task (IGT), which involves selecting one out of a set of four decks with higher and lower payoffs/losses (Bechara et al., 1994; Dunn et al., 2006). More specifically, IGT assesses the extent to which an individual can develop a successful strategy in decision-making, requiring a different assessment strategy for the start and the later stages of the task (Brand et al., 2007).

Previous studies have shown that most participants can develop a strategy in choosing decks that lead to limited losses (Bechara et al., 1997; Maia & McClelland, 2004). The task has also been instrumental in providing empirical support for the somatic marker hypothesis, a theoretical framework for explaining the neural basis of emotions influencing decision-making (Bechara et al., 2005; Naqvi et al., 2006). Desmeules et al. (2008) reported individual differences in performance on the task, and suggested a high sensitivity to gains or losses from two perspectives: scalar multiplication and valuation by emotion. Although initially used to evaluate decision-making impairments among individuals with neuropsychiatric disorders and addictions (Bechara & Damasio, 2002; Cavedini et al., 2002), the IGT also shows variety in abilities within populations not suffering from these disorders (Giustiniani et al., 2015), suggesting IGT performance can be affected by many different physiological and psychological (behavioral) phenomena (Giustiniani et al., 2019).

However, no previous study has addressed some of the key reasons underlying individual differences in decision-making performance that is observed in this task among the general population (Dunn et al., 2006). König (2021) reported that younger adults were more risk averse than their older counterparts in the IGT, and Simonovic et al. (2017) showed that participants practicing reflective thinking were more likely to develop appropriate IGT strategies, indicating that religiosity may mediate IGT performance. Therefore, given these findings, the first hypothesis (H_1_) addressed in the present study was: *Different individual religiosity levels will affect IGT performance within cohorts with similar financial risk-taking perceptions*.

Another widely used task for evaluating risk-taking is the Balloon Analogue Risk Task (BART; Lejuez et al. (2002)). In this task, participants are asked to inflate a balloon, with longer inflation times leading to higher profits but also higher likelihood of the balloon bursting and participants losing their earnings. The number of pumps before the balloon bursts is randomized among trials. BART performance has, among others, shown a good correlation with real-world risk-taking among adolescents (Lejuez et al., 2002) and distinguishes risk-taking behavior between age groups (Wilson et al., 2022), with König (2021) identifying younger participants being more risk-seeking when using the BART compared to older participants. However, it has been argued that the BART suffers from confounding bias, which reduces its ability to determine the extent to which participants are risk-seekers (De Groot, 2020). This indicates that the BART is more a measure of decision-making under uncertainty (De Groot & Thurik, 2018).

The BART also assesses propensity for risk-taking, but from the start of the task (Lejuez et al., 2002). Schürmann et al. (2019) used the BART to investigate the association between risk perceptions and risk-taking perception/behavior in a dynamic environment. The results showed the significant role that risk perceptions play in risky behavior and potentially can be used to improve an individual’s ability to identify real-world risk-takers. Also using the BART, Lauriola et al. (2014) reported individual differences in risky decision-making environments, related to differences in individuals’ sensation seeking trait, with higher impulsivity on the BART related to higher levels of sensation seeking with a low to moderate effect size.

A study by Chan et al. (2014) also showed that BART performance is influenced by exposure to texts including references to religious objects or divinities. Kupor et al. (2015) showed that individuals being reminded about a divine entity increased risk-taking on the BART, who also showed lower regard to divinity in the case of negative BART outcomes. Consequently, it can be argued that the BART can provide insight into the relationship between religiosity and risk-taking in situations with high uncertainty, and the extent to which religiosity influences decision-making in these situations. Based on this, H_2_ was: *Religious individuals will show different risk-taking behavior during the BART compared to non-religious individuals.*

Other tasks for evaluating risk-taking behavior include the Mixed-Gamble Loss-Aversion Task (Tom et al., 2007). The MGLAT can determine if decision-making during situations which carry risk are based on motivation (expected utility [Becker, 1968]) or conflict (utility distance) and therefore provide insights into the behavioral mechanisms that drive an individual’s decision-making, aligning with the theory of intuitive and reflective judgement (Kahneman, 2011; Pornpattananangkul et al., 2019). Previous research has argued that religiosity may (i) influence expected utility (Bhuian et al., 2018), and (ii) reduce stock market participation (Xu et al., 2022). Therefore, H_3_ was: *Individual differences in religiosity levels will affect risk-taking in the MGLAT among cohorts with similar risk-taking perceptions.*

### Electrophysiological Aspects of Financial Risk-Taking and Faith

While literature focusing on the association between religiosity and FRT have made empirical contributions to a very complex problem hard to measure, a more objectively driven approach may provide further context and help address some of the contradictions within it. A plausible approach towards achieving this is to bridge the objective measures that focus on religiosity and FRT (e.g., EEG), and the subjective measures that focus on religiosity and FRT (self-report accounts). This approach may suppositionally identify explorative theoretical grounds, characterized by their own set of complex empirical challenges. The first recognizable barrier is the neurological correlates that underpin religiosity.

While several studies have looked at differences in EEG activity for either religion (Imperatori et al., 2020; Rim et al., 2019; Tenke et al., 2017), or reward feedback (P3, feedback-related negativity -FRN) and other event-related potential characteristics in risk-taking/gambling (Balconi & Angioletti, 2022; Dyson et al., 2018; Giustiniani et al., 2015; Pornpattananangkul et al., 2019), as far as the present authors are aware, the combined effect of religion and risk-taking on EEG activity has not previously been investigated. However, the effect of religiosity on EEG activity is an important factor because a participant’s rating of religion and personal religiosity affects their perception to and acceptance of gambling and risk-taking. This may be an important confounder which affects results in EEG assessment on gambling behavior and risk-taking. Therefore, H_4_ was: *Differences in FRT attitudes and religiosity will affect the amplitude of time-domain electro-encephalographic (EEG) signals (P3 and FRN) measured during decision-making task feedback.*

## Methods

Thirty-seven participants (14 male, 23 female) were recruited from the general population through posters displayed around the first author’s university campus and postings on social media (*Reddit, LinkedIn*). The participants’ attitude toward financial risk-taking was assessed using a self-devised scale comprising seven items (Table 1), rated from 1 (*strongly disagree*) to 7 (*strongly agree*). These questions were selected such that they provide an idea about the likelihood/intention of a participant to engage with high-risk financial events. Questions combine items from household financial risk-taking questionnaires, such as the likelihood to gamble and household financing (fixed income vs. investing) (Wong & Carducci, 1991), and questions related to risk-taking among financially literate individuals who anticipate engaging with investment (e.g., stock markets [Lampenius & Zickar, 2005]). Similar questions have also been used in relating investment management with personality types (Lauriola & Levin, 2001; Mayfield et al., 2008). Participants’ level of risk-taking was determined based on median response values over all seven questions, with participants having a median equal to or below 3.0 considered risk-averse, and participants having a median equal to or above 5.0 being considered risk-taking. Participants with values between 3.0 and 5.0 were considered risk neutral. The FRT-questionnaire had a Cronbach alpha score of 0.729.

**Table 1:** List of items assessing financial risk-taking among participants with ratings

Financial Risk-Taking (FRT) statements.	Ratings						
I like to gamble often.	Strongly Disagree	Disagree	Slightly Disagree	Neither Agree nor Disagree	Slightly Agree	Agree	Strongly Agree
	1	2	3	4	5	6	7
I like to invest in risky ventures.	Strongly Disagree	Disagree	Slightly Disagree	Neither Agree nor Disagree	Slightly Agree	Agree	Strongly Agree
	1	2	3	4	5	6	7
I like to deal with uncertainty.	Strongly Disagree	Disagree	Slightly Disagree	Neither Agree nor Disagree	Slightly Agree	Agree	Strongly Agree
	1	2	3	4	5	6	7
I often like to reinvest even if losses can occur.	Strongly Disagree	Disagree	Slightly Disagree	Neither Agree nor Disagree	Slightly Agree	Agree	Strongly Agree
	1	2	3	4	5	6	7
I prefer investing in one investment rather than diversifying.	Strongly Disagree	Disagree	Slightly Disagree	Neither Agree nor Disagree	Slightly Agree	Agree	Strongly Agree
	1	2	3	4	5	6	7
I prefer risky investments over fixed income.	Strongly Disagree	Disagree	Slightly Disagree	Neither Agree nor Disagree	Slightly Agree	Agree	Strongly Agree
	1	2	3	4	5	6	7
I like to make more money.	Strongly Disagree	Disagree	Slightly Disagree	Neither Agree nor Disagree	Slightly Agree	Agree	Strongly Agree
	1	2	3	4	5	6	7

Religiosity was assessed using the five-item Centrality of Religiosity Scale (Huber & Huber, 2012). This scale includes questions about the frequency of praying and joining religious services, as well as the extent to which someone believes a divine entity exists. All items (e.g., *“How often do you take part in religious services?”*) are rated on a five-point scale before calculating overall religiosity levels. Religiosity levels were assessed using thresholds suggested by Huber and Huber (2012), taking the average scores of all five questions, and defining an individual as non-religious (average below or equal to 2.0), religious (2.1 to 3.9) or very religious (equal to or above 4.0).

### Risk-Taking Tasks and EEG Measurements

EEG data were collected from participants undergoing four decision-making tasks. These were the Balloon Analogue Risk Task (BART), Iowa Gambling Test (IGT), Mixed-Gamble Loss-Aversion Task (MGLAT) (Bechara et al., 2005; Lejuez et al., 2002; Pornpattananangkul et al., 2019), alongside a task based on the MGLA, but with participants informed if they had won or lost money after every round (hereafter: MGLA-Success Task [MGLAST]). The experiment order was randomized between participants using a Latin square design. EEG data were collected using a 20-electrode EEG system (Enobio20, Neuroelectrics, Spain), with electrodes located according to the standard 10–20 system. All tasks were written and controlled through MATLAB (R2020a, Natick, USA) and the PsychToolbox-3 add-on software (Brainard, 1997; Kleiner et al., 2007; Pelli, 1997).

During the BART, participants were asked to inflate a balloon such that they would gain as much profit as possible while avoiding the balloon bursting. The BART experiment was adapted in two ways from standard methods (Lejuez et al., 2002). More specifically, (i) participants only pressed a button to start and stop the inflation, and (ii) a color code was provided distinguishing three types of balloons based on their risk for exploding (green = low risk, amber = medium risk, red = high risk). Participants were not informed about color coding before the experiment. The experiment also included a set of rounds where no profit could be made (grey balloons) and a set of rounds where a fixed profit was made, because the balloon automatically inflated to a concentric circle surrounding it. Every participant completed 100 rounds. EEG data were recorded throughout the task. However, for calculating coherent and grand averages over rounds in which money was made versus those where money was lost, a MATLAB trigger was sent to the EEG data collection software at the time the participant banked their profit, or the balloon burst. This was followed by a two-second delay before starting the next round. Apart from EEG data, inflation time, balloon risk-level, and money won/lost were recorded for each round.

The IGT experiment employed a modified IGT in which participants pressed a button to decide to play or pass based on an automatically selected deck (Cauffman et al., 2010). Once a deck was selected, the participant received four seconds to decide. A null decision was recorded if participants did not decide within this interval. After each round, the participant was shown the result of their decision for two seconds. Null decisions were shown as “passed”. As with the standard IGT, two decks were high-risk/high-profit and two decks were low-risk/low-profit (Bechara et al., 2005). Participants received information about money gained/lost immediately after each of the 120 rounds. EEG data were recorded throughout the experiment, with triggers sent to the EEG system when participants were shown the result of each round. Time before deciding, result, amount of money gained/lost, and deck selected were recorded for each round.

For the MGLAT and MGLAST, 81 win-loss combinations were developed, with possible wins ranging from £1 to £9 (steps of £1) and losses ranging from £0.5 to £4.5 (steps of £0.5), leading to 61 epochs in which the win/loss ratio is above 1. Combinations were set up such that each win-loss combination occurred once per participant, with all combinations randomized between different participants. For each round, participants were first shown how much they could win that round. Participants were given as much time as needed (and at least one second) to memorize this value. After a two-second delay in which participants were asked to focus on a plus (+) sign, participants were shown how much they could lose. Again, participants were given at least one second to memorize the amount and, after another two-second delay, were asked if they would like to take the gamble or not by pressing a keyboard button. For the standard MGLAT, there was a one-second delay before the next round started. For the MGLAST, participants were shown the result of their decision for at least two seconds, after which they continued to the next round. Participants were made aware beforehand that, for each round, they had a 50% chance of winning. EEG recordings were measured throughout the task, with triggers sent at the time the “loss” amount appeared on the screen. For the MGLAST, a second trigger at the time participants were informed about the result for each round was added. For each round, decision, time for deciding, and the amount of money won/lost were recorded.

### EEG Data Processing

The BART EEG data were initially filtered using a 501st order finite-impulse response bandpass filter with cut-off frequencies at 0.01 and 100 Hz. This was followed by re-referencing signals to the average over all electrodes. This was followed by a second band-pass filtering step with cut-off frequencies at 0.1 and 30 Hz. EEG data were then epoched from 200ms before till 800ms after trigger timestamps indicating the end of individual rounds. Epochs in which no profit could be recorded or fixed profits were then discarded, leaving a total of 86 epochs per participant. These epochs underwent baseline correction based on the average EEG amplitude measured in their first 200ms. Epochs reaching values above 100µV were considered noise and discarded. Coherent averages, P3 amplitude at Pz, and FRN amplitudes at Cz and Fz were calculated after separating profit banked and balloon burst epochs. P3 was measured as the peak absolute amplitude within the interval 300ms–450ms after trigger onset. FRN was measured as the peak absolute amplitude within the interval 150ms–300ms after trigger onset. IGT EEG processing followed a similar approach to BART, with the exception of epochs being separated in groups in which participants had won money versus lost money.

MGLAT data processing followed an event-related potential (ERP) procedure adapted from Pornpattananangkul et al. (2019). EEG data were bandpass-filtered and re-referenced to the average electrode as before. Data were epoched from 200ms before to 3s after the loss-screen trigger, with detrending performed on a window of 2s before until 4s after the trigger. Baseline correction for each epoch was performed by subtracting the average amplitude of the first 200ms of each epoch. Epochs with amplitudes above 75µV were discarded. Epochs were investigated overall, as well as for rounds with a win/loss ratio above and below 1. For MGLAST, additional processing involved extracting epochs from 200ms before until 800ms after triggers identifying when results of decisions were shown to the participant. P3 and FRN amplitudes were measured as described above for the IGT and BART experiments.

### Data Analysis

EEG amplitudes were analyzed using pairwise *t*-tests with Tukey correction, comparing P3 and FRN amplitudes for BART data of “profit banked” against “balloon burst” rounds, and for IGT and MGLA-success data comparing “won” versus “lost” rounds.

Multivariate analysis of variance (MANOVA) was then conducted to determine the effect of FRT and/or religiosity on EEG (P3/FRN) activity and task (IGT/BART/MGLAT/MGLAST) performance indicators. Before applying MANOVA, multi-collinearity was assessed through variance inflation factor (VIF < 5), coefficient of determination (*R*^*2*^ < 0.99) and eigenvalue condition index (< 15), If any of these thresholds were violated, the independent variable was removed from further analysis. Moreover, equality of covariance matrices among different variable groups was evaluated using Box’s test. If this showed high significance (*p* < 0.01), Pillai’s trace test was used to assess significance within the MANOVA test. In other cases, Wilk’s lambda was used (Tabachnick & Fidell, 2020).

Further evaluation involved custom hypothesis tests determining if significant contrasts occurred between different religiosity/FRT groups. In cases where these were found, univariate tests on estimated marginal means of individual indicators were performed using ANOVA with post-hoc pairwise comparison after Tukey correction. All tests were considered significant at a 0.05 alpha level.

## Results

Based on answers to FRT-related questions, 29 participants were classed as risk-averse (78%), five risk-neutral (14%), and three risk-taking (8%). From a religiosity perspective, 14 were classed as non-religious (38%), 13 as religious (35%), and 10 as very religious (27%).

### Iowa Gambling Task

Paired *t-*tests showed significant differences in P3 amplitudes at Pz and FRN amplitudes at Fz and Cz when comparing “won” versus “lost” grand averages (*p* < 0.001). From a performance perspective, average decision-making times over all participants changed significantly (Friedman ANOVA, *p* < 0,001), with participants taking significantly less time to decide during the second, third and fourth set of 30 rounds compared to the first set. Similarly, variance in decision-making time varied significantly between the first and other sets (Friedman ANOVA, *p* < 0.001). No significant differences were found in play ratios nor profit made among sets. When comparing different decks, no significant differences were found in decision-making time or play ratios. Profits made varied significantly between high-risk and low-risk decks (ANOVA, *p* < 0.001).

MANOVA on EEG signals showed no significant effect of FRT (Pillai’s trace, *p* = 0.325), religiosity (Pillai’s trace, *p* = 0.333), or the interaction between religiosity and FRT (Pillai’s trace, *p* = 0.052). However, there was a significant effect of FRT on performance for one of the high-risk high-gain decks (*p* = 0.033). Contrast analysis showed that this was driven by a significant contrast in total profit made between risk-averse and risk-taking individuals (*p* = 0.008). ANOVA showed a significant difference in total profits (*p* = 0.025), with risk-taking participants showing significantly higher losses incurred compared to risk-averse individuals (*p* = 0.028).

### Balloon Analogue Risk Task

P3 and FRN amplitudes were significantly different between epochs in which participants won (profit banked) versus lost (balloon burst) (paired *t*-tests, *p* < 0.001). MANOVA showed a significant effect of FRT (*p* < 0.001), religiosity (*p* < 0.001), and the interaction between FRT and religiosity (*p* < 0.001) on BART EEG parameters. From a religiosity perspective, this was caused by significant contrasts in FRN amplitudes at Fz and Cz between non-religious and religious participants, alongside significant contrasts in FRN amplitude at Fz between religious and very religious participants. Pairwise tests showed a significantly higher FRN amplitude at Fz for “won” epochs among non-religious individuals compared to religious individuals (*p* = 0.008) and very religious individuals (*p* < 0.001), and a significantly higher amplitude in religious individuals compared to very religious individuals (*p* = 0.03). Similarly, FRN amplitude at Fz for “lost” epochs was significantly higher for non-religious versus religious individuals (*p* < 0.001) and very religious individuals (*p* < 0.001). FRN amplitude at Cz was also significantly higher for non-religious compared to religious individuals (*p* = 0.005) and very religious individuals (*p* = 0.013) for “won” epochs. For FRT, no significant contrasts between risk-taking levels were observed, but pairwise comparison showed significantly different FRN amplitudes at Fz for “won” epochs between risk-taking and risk-averse individuals (*p* < 0.001) and risk-neutral individuals (*p* = 0.007). Within risk-neutral individuals, significant differences were found between non-religious and religious individuals (*p* = 0.002), and non-religious and very religious individuals (*p* < 0.001).

Significantly different FRN amplitudes were also found at Fz for “lost” epochs between risk-neutral and risk-averse individuals (*p* < 0.001) and between risk-neutral and risk-taking individuals (*p* < 0.001). Within risk-neutral individuals, non-religious individuals significantly differed from religious individuals (*p* < 0.001) and very religious individuals (*p* < 0.001).

Risk-averse individuals also showed significantly different means of FRN amplitudes for “won” epochs at Cz compared to risk-neutral individuals (*p* = 0.023). Within risk-neutral individuals, non-religious individuals significantly differed from religious individuals (*p* = 0.003) and very religious individuals (*p* = 0.007).

The interaction between religiosity and FRT showed a significant effect on performance during low-risk BART rounds (*p* = 0.017), but no significant contrasts were found. Pairwise comparisons showed that, within the religious cohort, risk-neutral individuals had significantly longer average inflation time intervals compared to risk-averse individuals (*p* = 0.031) and risk-taking individuals (*p* = 0.044).

For medium-risk BART rounds, significant effects were found for religiosity (*p* = 0.039) and the interaction between religiosity and FRT (*p* < 0.001). The variance in inflation time differed significantly between very religious and religious individuals (*p* = 0.008), and very religious and non-religious individuals (*p* = 0.024) within the risk-neutral cohort.

### Mixed-Gamble Loss-Aversion Tasks

No significant differences were found between participants or sub-participant cohorts for the MGLAT and MGLAST for religiosity and FRT perspectives. However, significant effects were found regarding MGLAT average reaction time (Kruskal-Wallis, *p* = 0.021), and MGLAT winnings, average and variance of reaction time for rounds where the win/loss ratio was above 1 (Kruskal-Wallis, *p* = 0.047) when comparing individuals who performed MGLAT before versus after MGLAST. Overall MGLAST average (*p* = 0.034) and variance of reaction time (p = 0.006) also differed significantly between individuals who completed the MGLAT before versus after the MGLAST.

## Discussion

The present study investigated the extent to which financial risk-taking (FRT) perspectives and religiosity influenced an individual’s performance on financial decision-making tasks under risk and/or uncertainty. It further investigated the potential to measure this interaction using objective (EEG) assessments. Religiosity and FRT perspectives were assessed via the Centrality of Religiosity Scale (Huber & Huber, 2012) and a self-devised FRT questionnaire, respectively.

Previous research has shown controversy over the likelihood of religious individuals taking morality-abiding risks compared to non-religious individuals, including circumstances in which risk-taking was affected by direct or indirect religion-related messages (Chan et al., 2014; Gervais et al., 2020; Kupor et al., 2015). More broadly, it has been argued that higher levels of religiosity, or more frequent interaction with reflective thinking/spirituality (Simonovic et al., 2017) increase the likelihood of FRT, potentially through similar principles found in religious coping strategies (Kim et al., 2018) and reciprocal altruistic behavior encountered in other aspects of religious beliefs and practices (Bulbulia, 2004; Hobson et al., 2021; Trivers, 1971).

The present study found that religiosity and FRT may affect performance on BART in a complex interaction, but not performance on IGT and MGLA tasks. However, the findings did show that different perceptions on religiosity and FRT were related to different brain activity changes, verified objectively through EEG analysis of data collected during task performance. There now follows a discussion of findings according to each of the four hypotheses.

### Effects of Religiosity and FRT Perspectives on IGT Performance

Results suggested there was no relationship between religious orientation and the development of risk-averse strategies during the IGT. Therefore, H_1_ was not supported. Individuals who considered themselves to be risk-averse lost less money on high-risk decks, but this was not due to a discrepancy in play ratios compared to risk-taking and risk-neutral individuals.

It is often assumed that higher levels of religiosity are indicative of increased time spent on reflective thinking, and as such, results on IGT performance for highly religious individuals would be expected to align with those that engage more often with reflection and other forms of spirituality (Simonovic et al., 2017). This did not appear to be the case in the present study. The measure of religiosity used, which extended beyond time spent reflecting (praying) by including, for example, belief in the existence of and life intervention by a divinity, did not significantly affect IGT performance, and therefore the ability to develop appropriate strategies for IGT. Moreover, the present study did not find an effect for religious morality on reduced risk-taking. These findings align with the work by Gervais et al. (2020), further contradicting that religiosity, or reference to religiosity, negatively affects risk-taking behavior.

### Effects of Religiosity and FRT Perspectives on the BART

The present study showed an interactive effect of FRT perspectives and religiosity on BART performance. Therefore, H_2_ was supported. More specifically, when individuals were close to risk-neutral, religiosity influenced doubt in deciding the amount of risk that can be taken in situations with high uncertainty (medium-risk balloons). Compared to bursting low-risk (higher certainty of not bursting) and high-risk (higher certainty of bursting) balloons, very religious, risk-neutral individuals appeared to converge more to extreme behaviors (either short inflation or long inflation time) when the chance of the balloon bursting was difficult to judge. Although not tested, this variation may be caused by results in previous rounds, similar to the win-stay-lose-switch (WSLS) heuristic observed in investment decision-making (Weiss-Cohen et al., 2022). Other reasons may be individuals not understanding the difference between different balloon colors and therefore failing to develop an appropriate strategy overall, or different emphasis of religiosity (praying, belief in divine intervention) affecting risk-taking decisions differently. In essence, the present study agrees with and extends previous analysis provided by Kupor et al. (2015) that the BART can provide a link between religiosity within situations with high uncertainty, especially among individuals with no strong incentive towards or against risk-taking.

### Effects of Religiosity and FRT Perspectives on MGLA Tasks

Although previous research has suggested a potential effect of religiosity on expected utility (Bhuian et al., 2018), which can be measured using an MGLA task (Pornpattananangkul et al., 2019), the present study showed no significant effects of religiosity or FRT perspectives on MGLA performance. Therefore, H_3_ was not supported. However, an effect of chronology between the MGLAT and MGLAST was observed. Individuals performing the MGLAT after the MGLAST showed a shorter average reaction time after receiving a cue for decision-making. MGLA average and variance of reaction time, as well as profit made, for rounds with a win/loss ratio above 1 also decreased. With the MGLAST, the reaction time average and variance increased for individuals who performed MGLAT after the MGLAST. These results mostly appear to indicate increased familiarity with the task.

### Measuring Religiosity and FRT Perspectives Using EEG While Performing Decision-Making Tasks

Several studies have shown variations in frequency-band EEG activities between religious and non-religious individuals (Imperatori et al., 2020; Rim et al., 2019; Rosmarin et al., 2022; Tenke et al., 2017). Simultaneously, neural bases underlying variation within financial risk-taking have been suggested (Kuhnen & Knutson, 2005; Wu et al., 2012; Zheng et al., 2020) and measured with EEG. These bases have been demonstrated by investigating both frequency-domain perspectives of EEG data (Giustiniani et al., 2015; Leota et al., 2023; Pornpattananangkul et al., 2019); and time-domain event-related potentials (Gu et al., 2018; Pornpattananangkul et al., 2019; Zheng et al., 2020).

Religion and spirituality are often considered a potential part of behavioral therapy, whether it is problem gambling (or, more generally, problematic financial risk-taking behaviors [Gavriel-Fried et al., 2020; Griffiths et al., 2016]) or other forms of addictive behavior (Kupor et al., 2015; Rodda et al., 2018), as well as behavioral education (Holt et al., 2009). At the same time, religion is possibly considered by risk-takers (or those addicted) as a safety net against harm (Chan et al., 2014; Clarke et al., 2006). These effects, alongside the possibility of religion itself bearing problematic outcomes (Taylor, 2002), indicate that an understanding of changes in EEG activity will improve understanding of the complex effect of religiosity on (financial) risk-taking. In the present study, attention was paid to time domain-based variation in EEG activity upon receiving feedback in financial decision-making tasks.

EEG data collected during the IGT showed a significant difference in amplitude between rounds where positive (“won”) versus negative (“lost”) feedback occurred, which is expected based on previous work evaluating the effects of negative feedback on brain activity (Santesso et al., 2012). This effect was not found in the MGLAST, potentially due to losses being limited and therefore not inducing a strong response. Previous studies have shown that MGLA tasks may be useful in detecting different levels of expected utility and utility distance (Pornpattananangkul et al., 2019). The BART results also showed some level of sensitivity against different religiosity and FRT levels. These results concur with previous studies showing an effect on ERP P1 amplitudes in BART tasks with increasing levels of risk (Gu et al., 2018), in the sense that variations in willingness to take financial risks may have a physiological underlying mechanism, which is potentially driven by or co-localized with effects related to exposure, and belief in (and practice of) religious/spiritual rites. Therefore, H_4_ was partially supported.

Overall, the mediating effect of religiosity on FRT remains complex. One possible reason is the broadness of the religiosity concept, which may lead to different interpretations and perception of risk (Gervais et al., 2020; Kupor et al., 2015). Previous work has suggested three dimensions to religiosity (belief, experience, and ritual), may affect perceptions on life and life events differently (Tan & Vogel, 2008). Although not tested here, arguments could be made that individuals experiencing religiosity through perceptions that a divinity intervenes in their life may look differently to those who join religious services frequently, with the former believing divine entities may provide a cushion and the latter paying more attention to religiously moral behavior in day-to-day activities. This was also reported in Williams et al. (2022) and the findings indicated that supernatural aspects of religiosity predicted risky-taking behavior while social and ritualistic aspects of religiosity predicted risk-aversion. Therefore, the multidimensionality of religiosity may warrant further and specificized investigation despite the usefulness of religiosity as an overarching construct and measure in FRT studies. Hypothetically, stronger belief in supernatural facets of religious faith, such as divine intervention may provide a possible avenue for exploring the ambiguity in the dichotomy between religious individuals who are risk-averse due to religious reasons and religious individuals who are conversely risk-takers due to potentially religious reasons (Binde, 2007; Lam, 2006; Mutti-Packer et al., 2017). Moreover, religious tolerance or intolerance towards financial risk-taking is complex and influenced by the varying constructs of morality regarding financial risk-taking as defined by different religious traditions and interpretations of theological doctrine by specific religious institutions (Coleman, 2016; Hassanat & Al Tarawneh, 2015). In the present study, non-significant differences in experiencing religiosity were observed within the religious population. Future studies should focus on the effects of different aspects of religiosity on FRT with consideration for the multidimensionality of religious experiences, as well as the nuances of religious morality surrounding financial risk-taking behavior.

## Conclusion

The present study investigated the potential interaction of religiosity and perspectives on financial risk-taking among individuals based on decision-making task performance and EEG activity measuring the effects of feedback against these tasks. The interaction is driven by the idea that financial risk-taking and religiosity may both be driven by religious coping strategies and reciprocal altruistic concepts. The study showed that BART performance, but not MGLA tasks or IGT performance, may be affected by an interaction of FRT perspectives and religiosity. This interaction can also be observed objectively within EEG data. Overall, while religiosity and FRT may not significantly influence IGT and MGLA performance, and interact with BART in a complex way, physiological reaction towards feedback after BART performance appears strongly affected by religiosity and FRT perspectives.

## Data Availability

Data will be made available upon reasonable request.
